# Dry needling for spine related disorders: a scoping review

**DOI:** 10.1186/s12998-020-00310-z

**Published:** 2020-05-11

**Authors:** Matthew F. Funk, Aric J. Frisina-Deyo

**Affiliations:** University of Bridgeport College of Health Sciences, School of Chiropractic, 126 Park Avenue, Bridgeport, CT 06604 USA

**Keywords:** Spine, Neck pain, Back pain, Myofascial pain syndrome, Trigger point, Pain, Outcome assessment

## Abstract

**Introduction/Background:**

The depth and breadth of research on dry needling (DN) has not been evaluated specifically for symptomatic spine related disorders (SRD) from myofascial trigger points (TrP), disc, nerve and articular structures not due to serious pathologies. Current literature appears to support DN for treatment of TrP. Goals of this review include identifying research published on DN treatment for SRD, sites of treatment and outcomes studied.

**Methods:**

A scoping review was conducted following Levac et al.’s five part methodological framework to determine the current state of the literature regarding DN for patients with SRD.

**Results:**

Initial and secondary search strategies yielded 55 studies in the cervical (C) region (71.43%) and 22 in the thoracolumbar-pelvic (TLP) region (28.57%). Most were randomized controlled trials (60% in C, 45.45% in TLP) and clinical trials (18.18% in C, 22.78% in TLP). The most commonly treated condition was TrP for both the C and TLP regions. In the C region, DN was provided to 23 different muscles, with the trapezius as treatment site in 41.88% of studies. DN was applied to 31 different structures in the TLP region. In the C region, there was one treatment session in 23 studies (41.82%) and 2–6 treatments in 25 (45.45%%). For the TLP region, one DN treatment was provided in 8 of the 22 total studies (36.36%) and 2–6 in 9 (40.9%). The majority of experimental designs had DN as the sole intervention. For both C and TLP regions, visual analogue scale, pressure pain threshold and range of motion were the most common outcomes.

**Conclusion:**

For SRD, DN was primarily applied to myofascial structures for pain or TrP diagnoses. Many outcomes were improved regardless of diagnosis or treatment parameters. Most studies applied just one treatment which may not reflect common clinical practice. Further research is warranted to determine optimal treatment duration and frequency. Most studies looked at DN as the sole intervention. It is unclear whether DN alone or in addition to other treatment procedures would provide superior outcomes. Functional outcome tools best suited to tracking the outcomes of DN for SRD should be explored.

## Introduction/Background

Dry needling (DN) is a subcutaneous needle insertion technique using a fine, solid needle without anesthetic or injection [1]. DN can be distinguished from acupuncture, which although applied with a solid filiform needle, uses Traditional Chinese Medicine principles, [2] and also from other techniques that use injectable substances through a hypodermic needle [3]. When DN is combined with electrical stimulation of various frequencies and intensities, certain pain modulating neurotransmitters may be released [4–6]. DN has been further defined by the American Physical Therapy Association as “… a skilled intervention that uses a thin filiform needle to penetrate the skin and stimulate underlying myofascial trigger points, muscular and connective tissues for the management of neuromusculoskeletal pain and movement impairments. DN is a technique used to treat dysfunctions in skeletal muscle, fascia, connective tissue and diminish persistent peripheral nociceptive input, and reduce or restore impairments of body structure and function leading to improved activity and participation” [7].

Legge [8] observed that DN began when practitioners and researchers were looking for ways to treat muscular pain at tender points with injections and found that even without injections, DN could provide relief. Legge concluded that currently some practitioners and researchers specifically use DN solely to treat myofascial TrP, and that the majority of literature on DN refers to its use in the treatment of myofascial TrP. In the same article, Legge also pointed out that other practitioners support a larger use of DN for ligamentous, tendinous, articular and scar tissue disorders as well.

Current literature appears to support DN for the treatment of myofascial pain due to TrP [9]. Dommerholt postulated DN may help limit nociception from TrP and discussed the possible physiological mechanisms of pain relief with DN [4]. Dry needling in neck and back pain treatment has been evaluated previously. Ong [10] found no difference between DN and lidocaine injection for relief of neck and shoulder pain. In a systematic review and meta-analysis on the effectiveness of DN for low back pain (LBP) by Hu [11], DN was found more effective for reducing pain and disability than acupuncture, sham acupuncture, or other treatment. However, the effectiveness was equal to that of acupuncture at long term follow-up. Hu also concluded that the current evidence does not support the efficacy and safety of DN for LBP. A meta-analysis by Gattie [12] concluded there was very low to moderate evidence that DN to TrP yielded significant effect on functional outcomes in the short term, but was not better than other treatments in the long term.

A systematic review (SR) by Liu L et al. in 2015 [13] studied the effectiveness of DN or acupuncture versus wet needling with lidocaine injectate for both neck and shoulder pain and concluded that even though DN could be recommended for TrP in NP and shoulder pain, wet needling with lidocaine was more effective than DN in the medium term. Liu et al. included shoulder pain that could be from local sources (shoulder stuctures) that are not specifically a SRD and also included acupuncture, which we specifically excluded. Their conclusions may differ from future SR of studies looking at DN only for NP due to SRD only. Another SR by Liu et al. [14] looked at 11 RCTs using DN for LBP and found moderate evidence to recommend DN for TrP associated with LBP when used with other therapies. They concluded it was unclear whether DN was superior to other treatments for improving functional disability. Espejo-Antunez et al. [15] searched DN for myofascial trigger points and identified 90 articles, and then systematically reviewed 15 articles in areas ranging from the spine, knee and TMJ. They concluded that although there was some evidence to support use of DN for TrP for short term pain relief, further RCT using standardized procedures to identify TrP and apply DN are needed.

As mentioned above, the American Physical Therapy Association states DN can be used for the management of neuromusculoskeletal pain and movement impairments. Although DN treatment appears widely accepted for TrP, it is unclear which other painful conditions and movement impairments it is best suited for. For example, the use of DN for other painful conditions due to SRD as described by Murphy [16, 17] has not been summarized.

SRD that are not secondary to serious pathology have been described by Murphy as having four major pain generators. These are: disc derangement, radiculopathy, joint dysfunction and myofascial trigger points [16, 17]. Pain generators may occur alone or in any combination, and can often be differentiated through history and physical examinations. According to Murphy, TrP are common sources of axial and referred pain but typically form in response to pain from other sources, and once the primary pain source is relieved, the TrP will often resolve. However, in some cases, they persist and should be treated as well ([16], p. 247). Treatments recommended for TrP in Murphy’s Clinical Reasoning in Spine Pain (CRISP) protocols include manual pressure release techniques (digital or instrument assisted pressure), muscle lengthening techniques (i.e. post-isometric relaxation) and combined release and lengthening ([16], p., 248-9). Medical treatments available include oral and injected anesthetics, steroidal and non-steroidal anti-inflammatories, or botulinum toxin injection [18]. Murphy does not discuss DN as a potential treatment for TrP or other SRD.

The available meta-analyses (MA) and SR [10–15] only looked at specific subsets of data or were relatively generic in their inclusion criteria. Many of the papers discussed in this scoping review would not have been included in available systematic reviews or may have used DN treatment on disorders not specifically defined as SRD.

Therefore we sought to perform a scoping review to describe the DN research in the context of its use in all SRD, including spine-region TrP. Scoping reviews describe the extent of current literature on a subject and help to expose gaps that could be targets of potential future research. This scoping review seeks to identify research from case reports (CR), case series (CS), clinical trials (CT) and randomized controlled trials (RCT) published on DN treatment for patients with SRD diagnoses and to make evident where research is plentiful as well as lacking. We hope to add to the discussion of the role of DN in neuromusculoskeletal treatment to specifically include SRD. This review therefore will categorize which muscles or other structures have been used as treatment sites, whether treatment was directed at specific myofascial TrP only, or other named structures, the outcome measures studied and whether DN was considered effective.

## Methods

A scoping review of the literature on DN for SRD was conducted following the methodological framework as first outlined by Arksey et al. [19] and further refined by Levac et al. [20] and Peters et al. [21]. This framework, consisting of the five steps below, aims to elucidate the current state of the literature on the topic and highlight any gaps in the knowledge base.

### Step 1. Identifying the research question

What is the state of the current primary research literature on DN for patients with SRD? Specifically, which SRD diagnoses or conditions have been studied, and which specific structures have been treated in these studies? In addition, how many treatments have been used in the studies? Was DN the sole intervention or was it combined with other interventions, and finally, what outcome tools were used to determine effectiveness?

### Step 2. Identifying relevant studies

Two reviewers initially conducted independent searches in PubMed, AMED, CINAHL and Cochrane Library for relevant publications from 1990 through August 7, 2018, when the search period ended. National Library of Medicine Medical Subject Headings were created by combining “dry needling” (only, to limit the search to exclude acupuncture or other needle techniques), with terms that reflect conditions considered to be SRD. These SRD terms include general ones such as neck pain and back pain, regional spine terms such as cervical, thoracic, or lumbar, as well as pain-generating structures including myofascial, articular, nerve or disc. Using the subject heading terms listed in Table 1, each reviewer independently compiled a primary list of prospective studies to be reviewed. An additional search of PubMed for papers with the subject “dry needling AND trigger point” was done in order to capture any studies missed in the initial search strategy. Another search was performed in PubMed with the term “dry needling”, and papers from before 1990 were selected. The grey literature was independently searched utilizing Open Grey, Clinicaltrials.gov, Canadian Agency for Drugs and Technologies in Health, New York Academy of Medicine Grey Literature Report, University of York Centre for Reviews and Dissemination, National Technical Information Service and the U.S. Government Documents (Table 2). The reference lists of all included studies were reviewed to ensure a complete search was performed.

**Table 1:** Search terms

Cervical region	Thoraco-lumbar-pelvic region
DN and neck pain	DN AND (back pain OR sacroiliac OR spine)
DN and headache	DN AND disc AND (lumbar OR thoracic)
DN AND nerve AND (cervical OR neck)	DN AND (neur* OR sciatic*)
DN AND neur*	DN AND (joint OR articul*)
DN AND (joint or articul*)	DN AND disc
DN AND disc	DN AND paravertebral
DN AND myofascial	DN AND (thor* OR lumb* OR sacr* OR glut*)
DN AND trapezius	DN AND trigger point
DN AND cervic* muscle	
DN AND trigger point

**Table 2:** Grey literature sources and results

Database	Dry needling AND spine hits	Met Inclusion	Duplicates
Open Grey	1	0	0
ClinicalTrials.gov	2	0	0
CADTH	8	1	1
NYAM	0	0	0
CRD - York	20	6	6
U.S. Gov Docs	0	0	0
**Total**	**31**	**7**	**7**

### Step 3. Study selection

Each author independently retrieved papers from the primary list and eliminated titles as per the inclusion and exclusion criteria (as listed in Table 3). The authors compared results and any discrepancies were mediated through discussion. Each author then independently reviewed abstracts and further eliminated papers according to the inclusion/exclusion criteria.

**Table 3:** Inclusion and exclusion criteria

Inclusion Criteria	**Cervical region**: posterior from the superior nuchal line and mastoid to the spine of the scapula, anteriorly from the inferior border of the mandible to clavicle, and laterally to the acromion of the scapula. Pain and/or treatment within region defined above.
	**Thoraco-lumbar-pelvic (TLP) region**: posteriorly from the inferior border of the spine of the scapula to the coccyx, laterally to the mid-axillary line, inferiorly to the horizontal lower margin of the buttock at its junction with the thigh (gluteal fold). Pain and/or treatment within the region defined above.
Exclusion Criteria	Dry Needling not specified in title or abstract
	Pathological conditions, non-mechanical pain or secondary to other pathology
	Not English language
	Full paper available through university library
	Post-August 7, 2018
	Study types: non-experimental designs, narrative reviews, systematic reviews, meta analyses, protocols, reports of complications only, editorials, commentaries, patient brochures, poster summaries, letters to the editor
	Methodologies: non-human subjects, asymptomatic patients only, physiological responses only, treatment effects not reported, side-effects only reported without other clinical effects

### Step 4. Charting the data

The inclusion and exclusion criteria were reapplied to the selected full papers and the following data were collected: author, title, year of publication, study design, number of study subjects and spinal region. Other information was collected including the conditions treated with DN (and specific muscles when described), purpose of study, duration of study, intervention(s), comparison(s) as well as outcome measures used. Each study’s reported results were also collected.

### Step 5. Summarizing and reporting the results

Studies on DN for SRD were recorded on a Microsoft Excel® spreadsheet separately for the cervical (C) and thoracolumbar-pelvic (TLP) regions. Study types, including CR, CS, CT and RCT are presented in Table 4 as totals and percentages. The condition(s) treated are presented in Table 5. The muscle(s) treated, when specified, are presented in Tables 6 and 7. The number of treatments provided to subjects in each study can be found in Table 8. Table 9 presents the number of studies in which DN was the only treatment as well as those that combined DN with another treatment (DN+). The outcomes measured (such as VAS, ROM, patient reported outcomes) and the outcomes improved following treatment are presented in Table 10. These quantitative results were analyzed to reveal trends and gaps in the research for DN in SRD.

**Table 4:** Study type, number of papers (percent of total) per region

	Cervical	TLP
Randomized Controlled Trial (RCT)	37 (60)	10 (45.45)
Case Report (CR)	6 (10.91)	4 (18.18)
Clinical Trial (CT)	10 (18.18)	5 (22.73)
Case Series (CS)	2 (3.64)	3 (13.64)
**Total**	**55 (71.43% of studies)**	**22 (28.57% of studies)**

**Table 5:** Number of studies describing treatment per condition per region (percent of total)

	Cervical (55 studies)	TLP (22 studies)
myofascial trigger points	29 (43.28)	10 (31.25)
myofascial pain	14 (20.9)	3 (9.37)
neck pain	10 (14.93)	(3.12)
cervicogenic headache (HA)	3 (4.48)	
tension-type HA	1 (1.49)	
HA (type not specified)	1 (1.49)	
occipital neuralgia	1 (1.49)	
dizziness	1 (1.49)	
wry neck	1 (1.49)	
chronic whiplash associated disorder	1 (1.49)	
cervical radiculopathy	1 (1.49)	
reduced cervical mobility	1 (1.49)	
shoulder pain	1 (1.49)	
back pain	1 (1.49)	
muscle pain	1 (1.49)	
chronic low back pain (LBP)		6 (18.75)
mechanical LBP		3 (9.37)
thoracic pain		2 (6.25)
discogenic LBP with radiation		1 (3.12)
lumbar herniated disc		1 (3.12)
posterior thigh pain		1 (3.12)
lumbar spinal stenosis		1 (3.12)
sacroiliac and lumbar pain		1 (3.12)
ankylosing spondylitis		1 (3.12)
fibromyalgia		1 (3.12)
**Total conditions treated**	**67**	**32**

Some studies included treatment for multiple conditions

**Table 6:** Number of studies describing structure treated in the cervical region (percent of total)

	Cervical
Upper trapezius	35 (29.91)
Trapezius (site unspecified)	11 (9.40)
Levator scapulae	10 (8.55)
Suboccipital muscles	(5.13)
Cervical paravertebrals	(4.27)
Cervical multifidi	4 (3.42)
Semispinalis capitis	4 (3.42)
Middle trapezius	3 (2.56)
Supraspinatus	5 (4.27)
Splenius capitis	4 (3.42)
Infraspinatus	5 (4.27)
Sternocleidomastoid (SCM)	3 (2.56)
Posterior cervical (site unspecified)	2 (1.71)
Masseter	2 (1.71)
Temporalis	2 (1.71)
Spinalis capitis	1 (0.85)
Clavicular (SCM)	1 (0.85)
Splenius cervicis	1 (0.85)
Scalenes	2 (1.71)
Rhomboid	2 (1.71)
Teres minor	1 (0.85)
Thoracic multifidi	1 (0.85)
Posterior arch C1	1 (0.85)
Spinous process C2	1(0.85)
Acromioclavicular joint	1(0.85)
Unspecified cervical spinous processes	1(0.85)
Other miscellaneous non-muscular Structures	1(0.85)
Platysma	1(0.85)
Unknown	1 (0.85)
**Total conditions treated**	**117**

Some studies included treatment for multiple muscles

**Table 7:** Number of studies describing structure treated in TLP region (percent of total)

	TLP
lumbar multifidi	6 (8.45)
quadratus lumborum	9 (12.68)
gluteus medius	8 (11.27)
gluteus maximus	5 (7.04)
thoracic multifidi	2 (2.82)
thoracic paravertebrals	3 (4.23)
paravertebrals (unspecified)	2 (2.82)
piriformis	4 (5.64)
lower trapezius	2 (2.82)
latissmus dorsi	2 (2.82)
thoraco-lumbar iliocostalis	2 (2.82)
lumbar erector spinae	2 (2.82)
sacral multifidi	1 (1.41)
multifidi (unspecified)	1 (1.41)
psoas major	1 (1.41)
iliopsoas	2 (2.82)
gluteus minimus	3 (4.23)
gluteal muscles	1 (1.41)
tensor fasciae latae	2 (2.82)
hamstrings	1 (1.41)
rectus femoris	2 (2.82)
gastrocnemius	1 (1.41)
iliolumbar^a^	1 (1.41)
transforaminal epidural	1 (1.41)
lumbar paravertebrals	1 (1.41)
pelvic ligaments (unspecified)	1 (1.41)
ribs	1 (1.41)
ischial tuberosity	1 (1.41)
unspecified thoracic or lumbar spinous processes	1 (1.41)
miscellaneous non-muscular structures	1 (1.41)
segmental myotomal points throughout the spine and lower extremity	1 (1.41)
**Total conditions treated**	**71**

Some studies included treatment for multiple muscles

^a^iliolumbar specified as muscle treated

**Table 8:** Number of treatments per region

	Cervical	TLP
1	23	8
2	4	2
3	9	2
4	8	2
5	1	2
6	3	1
7	0	0
8	3	1
9	1	0
13+	0	1
variable	1	1
unknown	1	2
**Total**	**55**	**22**

**Table 9:** Number of studies with DN as sole intervention versus DN used with another intervention (DN+)

	Cervical (55 studies)	TLP (22 studies)
DN	34	10
DN+	24	13
**Total**	**58**	**23**

Some studies had multiple treatment arms

**Table 10:** Outcome tools used and improved with DN

	Cervical (55 studies)		TLP (22 studies)	
	No. studies	No. improved with DN^a^	No. studies	No. improved with DN^a^
Visual Analog Scale (VAS)	35	33	11	11
Pain Pressure Threshold (PPT)	31	28	7	7
Range of motion (ROM)	27	24	4	3
Short Form-36 (SF-36)	8	7	1	1
Numeric Pain Rating Scale (NPRS)	8	8	6	6
Beck Depression Index (BDI)	4	3	2	1
Oswestry Disability Index (ODI)	2	2	8	8
Self-rated improvement scale	1	0	1	1
Patient reported pain	2	2	2	2
SF-12 Physical	1	1	1	1
SF-12 Mental	1	1	1	1
Neck Disability Index (NDI)	13	13		
Medication use	5	5	2	2
Nottingham Health Profile (NHP)	3	2		
Dizziness Handicap Inventory (DHI)	2	2		
Headache index	2	2		
Symptom Severity Index (SSI)	2	2		
Verbal Pain Scale	2	2		
Brief Pain Inventory (BPI)	2	1		
Profile of Mood States (POMS)	2	1		
Beck Anxiety Index (BAI)	1	1		
Disabilities of the Arm, Shoulder and Hand (DASH)	1	1		
Geriatric Depression Scale Short Form (GDS-SF)	1	1		
Neck Pain Disability Index (NPDS)	1	1		
Northwick Park Neck Pain Questionnaire (Spanish)	1	1		
Nottingham Extended Activities of Daily Living Scale (NEADLS)	1	1		
Quadruple Visual Analog Scale (QVAS)	1	1		
Sympathetic skin response (SSR)^b^	1	1		
Assessment of change (11 point)	1	0		
Fall Index	1	0		
Hamilton Anxiety and Depression Inventory	1	0		
Global Rating of Change (GRoC)			4	3
Tampa Scale for Kinesiophobia (TSK)			2	2
US-imaging of multifidi			2	2
Functional Rating Index			1	1
McGill Pain Questionnaire (MPQ)			1	1
Northwick Park Neck Pain Questionnaire			1	1
Patient specific functional scale			1	1
Roland-Morris Disability Questionnaire (RMQ)			1	1
Sleep quality	2	2	2	2
Straight Leg Raise Test (SLR)			1	0
4 point patient subjective improvement scale			1	1
**Total (percent improved)**	**166**	**149 (89.76%)**	**63**	**58 (92.06%)**

Some studies used more than one outcome measure

^a^improvement as per author

^b^physiological response

## Results

### Relevant studies identified and collected

**Fig. 1:**
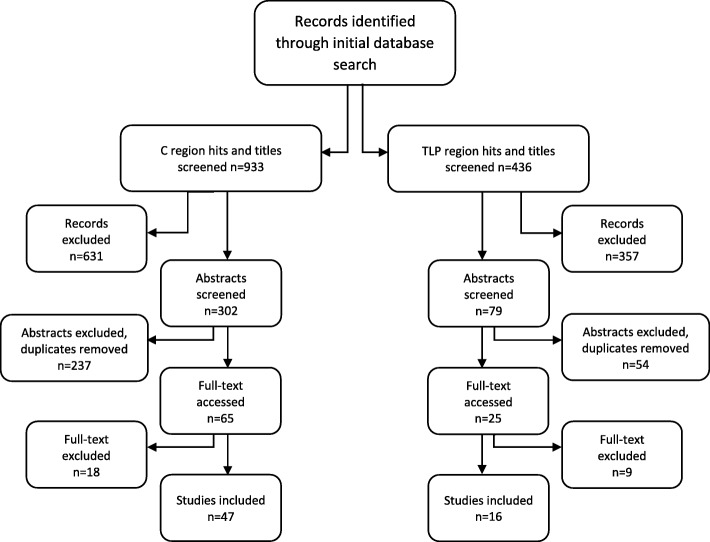
PRISMA flow diagram illustrating the records identified and those that were excluded and included during the initial literature search

Figure 1 presents the PRISMA flow diagram [22] illustrating the records identified and those that were excluded and included during the initial literature search. There were 933 titles screened in the C region and 436 in the TLP region. From the initial titles screened, 302 and 79 abstracts were reviewed from the C and TLP regions, respectively. Forty-eight studies were included in the C region and 16 in the TLP region for further analysis. The MeSH terms used during the literature search for the C region and TLP region are shown in Table 1. This search was completed in PubMed, AMED, CINAHL and the Cochrane Library. A secondary search of PubMed for “dry needling AND trigger point” yielded 294 total hits, of which 288 were excluded. Five studies involved treatment in the cervical region [23–27] and three in the thoraco-lumbo-pelvic region [23, 25, 28]. Two studies included DN treatments in both the cervical and TLP regions [23, 25]. Furthermore, an additional search for pre-1990 papers using PubMed and the term “dry needling” yielded 9 hits, of which four met criteria. Two papers were included that had treatment in the cervical region [29, 30] and three in the TLP region [30–32], with one providing treatment in both regions [30]. Table 2 depicts the grey literature search, the search term and databases searched, as well as the number of studies which met inclusion criteria. All studies that met inclusion criteria (1 from CADTH and 6 from CRD–York) were duplicates already found during the original database search. The inclusion and exclusion criteria utilized during the literature search are presented in Table 3. Additional file 1, (“Complete data as Tables 4.1 and 4.2”) contains each selected study’s reference number [33–95], lead author, and article title that are displayed in Tables 4.1 (Complete data, Cervical region) and 4.2 (Complete data, TLP region).

### Selected study parameters

Additional file 1 (Tables 4.1 and 4.2) also include research methodology, experimental subject population (n), study parameters, condition(s) treated with DN, any comparison intervention, the duration of study with number of treatment sessions, muscle(s) treated, whether DN was used alone, treatment in addition to DN, outcome tool(s) used, outcomes improved with DN, results and implications, whether DN was superior to other intervention, and additional comments.

#### Study design

Table 4 presents the number of studies and percent of total studies in the categories of RCT, CT, CR, and CS in both C and TLP regions. Fifty-five studies were found in the C region (71.43%) and 22 in the TLP region (28.57%). Most studies were RCT (60% in C, 45.45% in TLP) and CT (18.18% in C, 22.78% in TLP).

#### Intervention characteristics

Table 5 depicts the conditions treated per region as described in each study. A few studies investigated DN for multiple conditions. Treatment was provided for specific diagnoses as well as relatively non-specific conditions such as neck pain (NP), shoulder pain, back pain, muscle pain, thoracic pain, posterior thigh pain and sacroiliac plus lumbar pain.

In the 55 C region studies there were a total of 67 conditions treated, and in the 22 TLP region studies, 32 conditions were treated. The most commonly treated condition was TrP for both the C and TLP regions. There were 29 studies describing treatment for TrP in the C region (43.28%) and in the TLP region there were 10 (31.25%). The condition “myofascial pain” was studied in an additional 14 (20.9%) in the C region and 3 (9.37%) in the TLP region. Neck pain was described as the condition treated in 10 (14.93%) of the C region studies. One study applied DN to the C region for “back pain.” In the C region, cervicogenic headache (CGH) was described as the condition treated in 3 (4.48%) of studies, while tension-type headache (TTH), unspecified headache (HA), and occipital neuralgia were the target condition in one study each. Combining CGH, TTH, unspecified HA and occipital neuralgia, there were six studies (10.91%) on DN for all HA conditions.

In the TLP region, the most common condition treated was chronic LBP, found in 6 studies (18.75%), with mechanical LBP next at 3 (9.37%) and thoracic pain in 2 (6.25%). Discogenic LBP with radiation, lumbar herniated disc, posterior thigh pain and lumbar spinal stenosis were the targets of DN in one study each. One study applied DN to the TLP region for NP.

The particular structures treated with DN in the C region, when specified, are listed in Table 6. Most treatments in the C region were directed to muscle except for one study that did not specify the muscle treated, and a study that included DN treatment of bone or other non-muscular structures [30]. Of the 55 C region papers, DN was provided to 23 different muscles. There were a total of 117 treatments in the C region. The upper trapezius, trapezius, and middle trapezius combined for 49 of 117 total treatment sites mentioned (41.88%). Many studies performed treatments directed to multiple muscles.

Table 7 presents the 31 different structures treated with DN in the TLP region. There were a total of 64 muscle treatment sites, since again, some studies treated multiple muscles. The “iliolumbar” muscle was described as the target of DN in one study [94]. One study investigated a site that was not muscle, namely the effects of transforaminal epidural DN on lumbar spinal stenosis [80]. The quadratus lumborum, gluteus medius and lumbar multifidi were the most commonly treated muscles in the TLP region. Some muscles were not specifically described, such as “gluteal muscles” and “hamstrings.” The exact sites were not specified in two studies of DN treatment to paravertebral muscles [93, 95] and in one study of DN to multifidi [87]. Treatment to multifidi (lumbar, thoracic, sacral and unspecified), comprise 10 of the 64 TLP region treatment sites mentioned (15.62%).

Table 8 shows the number of DN treatments, per region, as described in each study’s Methods section. In the C region, there was just one treatment provided in 23 studies (41.82%), 2–6 treatments in 25 (45.45%), 7–9 treatments in 4 studies (7.27%) and none had 13 or more. One study varied treatment numbers per study arm and one did not describe the number of DN treatments provided. For the TLP region, one DN treatment was provided in 8 of the 22 total studies (36.36%), 2–6 in 9 (40.9%), one had between 7 and 9 treatments, and one had more than 13 treatments.

Table 9 provides the number of studies with DN as the sole intervention versus DN used with another intervention. There were 58 total investigations in the C region and 23 in the TLP region. About half (54.32%) of experimental designs had DN as the sole intervention.

### Outcomes measures and results reported

Table 10 shows the number of studies using different outcome assessment measures in the C and TLP regions along with those demonstrating improvement in outcome post DN as described by the study authors. For both the C and TLP regions, Visual Analogue Scale (VAS), Pressure Pain Threshold (PPT) and Range of Motion (ROM) were the most common outcomes. The most commonly used C region-specific outcome measure used was Neck Disability Index (NDI); Oswestry Disability Index (ODI) was the most common outcome measure in the TLP region. Most studies reported substantial improvement post DN in the measures studied. Improvement was reported in 89.76% of C region outcomes and 92.06% of the TLP region outcomes.

## Implications

### Spinal regions studied

It is estimated that 80% of the population will experience at least one episode of LBP [96]. Back pain, defined as below the 12th rib and not including the upper and mid-back [97], is the single leading cause of disability and imposes a very high economic burden worldwide. Neck pain lifetime prevalence is estimated at about 50% [98]. Although the incidence of LBP is greater than that of NP, only 28.57% of the included studies investigated DN for the TLP region, compared to 71.43% from the C region. More research should be done to establish the clinical relevance of DN for SRD below the C region, given the fact that LBP has greater population impact.

### Conditions studied

DN was often applied to muscle or myofascia regardless of the diagnosis. Most studies inserted the needle into muscle with exceptions being transforaminal epidural DN [80], pelvic ligaments and various bony landmarks, such as spinous processes [30]. Non-specific terminology was often used to describe conditions treated. For example, in some cases, non-specific diagnoses such as NP or chronic LBP were made and treatment rendered without a specific pain generator described. In addition, there were examples where the methodology did not specify the exact needle insertion location, only stating the general area or large muscle region, such as posterior cervical muscles, trapezius, paravertebral muscles and multifidi (unspecified) as shown in Tables 6 and 7. The exact sites of DN insertion were not included in all studies. Increased treatment site specificity may provide further clinical insight in future research.

Myofascial pain or TrP were the most commonly treated conditions in each region comprising 64.18% of the studies in the C region and 40.62% for the TLP region. The American Physical Therapy Association’s Educational Resource Paper on Dry Needling [7] defines DN as an intervention for neuromusculoskeletal pain and movement impairments such as TrP and other muscular and connective tissue disorders. A few studies, as shown in Table 5, evaluated DN for non-myofascial conditions. The APTA also states DN is a treatment option to reduce or restore impairments of body structure and function. Since we found very few studies investigating non-myofascial conditions, it is unclear what is meant by this recommendation. Only two studies in the C region specifically evaluated nerve-related pain (cervical radiculopathy, occipital neuralgia) and only two studies evaluated disc-related conditions (discogenic LBP with radiation, lumbar herniated disc). Murphy suggests that TrP are often secondary to other SRD [16]. It is unknown whether DN creates an important and lasting patient response if applied specifically to articular, disc or nerve structures or if DN is only effective when applied to TrP secondary to other conditions. Further investigation into the effects of DN applied directly to non-myofascial pain generators may provide further clinical insights.

### Treatment parameters

As seen in Table 8, most studies applied just one treatment before assessing the outcome. One could question if this mirrors clinical practice. Is it more common to apply multiple treatments, and at what frequency? The relative effectiveness may vary with different treatment numbers, frequency or duration. Specific investigation of optimal frequency and duration could be helpful for clinical practice. We found no studies that specifically investigated variable treatment numbers and frequencies for a given SRD. This is supported by Dunning et al. [1] who state “the optimal frequency, duration, and intensity of dry needling has yet to be determined for many neuromusculoskeletal conditions.” Future research may help determine a standard clinical treatment practice for different conditions.

### Interventions (DN alone or in combination)

Most studies looked at DN as a sole intervention while fewer had DN plus another intervention in the experimental design. It would be helpful to know if clinical outcomes are improved when DN is used as a component in a treatment plan consisting of multiple procedures and modalities. Regarding DN being used as a sole intervention in SRDs, it is unclear if this comports with common clinical practice. Future research that compares DN alone along with DN plus other treatments may help determine optimal treatment protocols.

### Outcomes

It appears that DN is beneficial for a number of conditions, as shown in Table 10. Most studies in the C region used pain scale ratings such as VAS, NPRS, verbal pain scale and QVAS. Also popular were PPT and ROM. In fact, 62.65% included pain scales, PPT and ROM within the 55 C region studies. The most popular functional outcome tools in the C region were the NDI, followed by the SF-36. Although studies investigating only physiological responses were excluded, one study used sympathetic skin response along with other outcomes [61]. Some outcomes such as NHP, BDI and DHI were used in four studies or fewer. In the TLP region VAS, PPT, ROM and NPRS accounted for 44.44% of the investigated outcomes. The most popular functional indices in the TLP region were ODI and GRoC, which combined for 19.05% of outcomes studied. As in the C region, there were other outcome measures used in a small number of studies such as BDI, MPQ, RMQ and TSK amongst others. Two studies looked at ultrasound imaging of the multifidi along with other outcomes studied [84, 86]. It is unknown whether some treatment effects of DN for SRD may be best demonstrated with particular functional indices.

Although scoping reviews do not critically appraise the quality of the literature, it appears most of the cited authors concluded that DN contributes to improved outcomes in many SRD. These outcomes were demonstrated regardless of diagnosis, number of treatments or patient population. Future studies that look at strict diagnostic and inclusion criteria, detailed treatment methods and most applicable outcome measures would be helpful in filling the gaps in the literature as it relates to the effectiveness of DN for SRD.

## Limitations

By limiting our title and abstract searches to DN (only), the authors recognize that papers describing other needle-based techniques that are not specifically called DN were excluded. Such studies may have data reported that could help in the overall understanding of the effects of various needling principles and techniques in treatment of SRD. In our understanding of SRD according to Murphy, there may be some conditions that were not included. However, studies investigating treatment for conditions that appeared to be emanating from the spine, and not secondary to a serious pathology such as cancer or infection, were included to the best of our ability. Furthermore, we limited our search to English language only, thus excluding studies in other languages that may have been sources of additional data.

## Conclusions

This scoping review demonstrates that for SRD, DN was primarily applied to myofascial structures for myofascial pain or TrP diagnoses, although other non-myofascial and non-specific diagnoses were also treated. Dry needling treatment to non-myofascial sites has been investigated primarily in extremity conditions [99]. There is currently little research on DN that specifically targets other SRD pain generators [16] including nerve roots, disc, tendons, ligaments, periosteum, scar tissue or fascia in SRD. It appears that many outcomes are improved regardless of diagnosis or treatment parameters. Most of the included studies applied just one treatment before assessing an outcome, which may not reflect common clinical practice. Further research is warranted to determine optimal treatment duration and frequency for SRD using DN. Most studies looked at DN as the sole intervention. It is unclear whether DN as a sole intervention or in conjunction with other treatments provides the best patient outcomes. The most commonly studied outcomes were pain rating scales, PPT and ROM while other patient reported outcomes were used less frequently. It is not known what functional outcome tools would be best suited to tracking the outcomes of DN for SRD.

## Supplementary information

**Additional file 1: Table 4.1.** Complete Data Cervical Region. **Table 4.2.** Complete Data TLP Region.

**Additional file 2.** Preferred Reporting Items for Systematic reviews and Meta-Analyses extension for Scoping Reviews (PRISMA-ScR) Checklist.

## Data Availability

All data generated or analysed during this study are included in this published article [and its supplementary information files].

## Abbreviations

AROM: Active range of motion
BAI: Beck anxiety index
BDI: Beck depression index
BPI: Brief pain inventory
BTA: Botulinum toxin-A
CADTH: Canadian Agency for Drugs and Technologies in Health
C-LF: Cervical lateral flexion
CLBP: Chronic low back pain
CNP: Chronic neck pain
CR: Case report
CRD-York: Centre for Reviews and Dissemination-University of York
DASH: Disabilities of arm, shoulder, hand
DDN: Deep dry needling
DHI: Dizziness handicap inventory
DN: Dry needling
DN+: Dry needling with additional intervention
EIS: Electrical intramuscular stimulation
EMG: Electromyography
FABQ: Fear avoidance beliefs questionnaire
FMS: Fibromyalgia syndrome
f/u: Follow-up
G: Group
GDS-SF: Geriatric depression scale-short form
GRoC: Global rating of change
HPPT: High-powered pain threshold
IASTM: Instrument assisted soft tissue mobilization
LBP: Low back pain
MET: Muscle energy technique
MPQ: McGill pain questionnaire
MPS: Myofascial pain syndrome
N/A: Not applicable
NEADLS: Nottingham extended activities of daily living scale
NDI: Neck disability index
NHP: Nottingham health profile
NP: Neck pain
NPDS: Neck pain disability scale
NPQ: Nonverbal personality questionnaire
NPRS: Numeric pain rating scale
NTIS: National Technical Information Service
NYAM: New York Academy of Medicine
ODI: Oswestry disability index
OMT: Orthopedic manual therapy
PENS: Percutaneous electrical nerve stimulation
POMS: Profile of mood states
PPT: Pressure pain threshold
PROM: Patient reported outcome measures
PT: Physical therapy, physiotherapy
QL: Quadratus lumborum
QoL: Quality of life
RCT: Randomized controlled trial
RMQ: Rolland Morris disability questionnaire
ROM: Range of motion
SCM: Sternocleidomastoid muscle
SCS: Strain-counterstrain
SF-36: Short form-36
SI: Sacroiliac
SLR: Straight leg raise test
SNAG: Sustained natural apophyseal glide
SSR: Sympathetic skin response
STT: Soft tissue therapy
TENS: Transcutaneous electrical nerve stimulation
TFL: Tensor fasciae latae
TrP: Myofascial trigger point
TSK: Tampa scale of kinesiophobia
US: Ultrasound
VAS: Visual analogue scale
WAD: Whiplash associated disorders
y.o.: Year old
